# Molecular Markers of Anticancer Drug Resistance in Head and Neck Squamous Cell Carcinoma: A Literature Review

**DOI:** 10.3390/cancers10100376

**Published:** 2018-10-10

**Authors:** Sandra López-Verdín, Jesús Lavalle-Carrasco, Ramón G. Carreón-Burciaga, Nicolás Serafín-Higuera, Nelly Molina-Frechero, Rogelio González-González, Ronell Bologna-Molina

**Affiliations:** 1 Research Institute of Dentistry, Health Science Center, Universidad de Guadalajara, Guadalajara 4430, JAL, Mexico; patologiabucal@live.com.mx; 2 Department of Research, School of Dentistry, Universidad Juárez del Estado de Durango, Durango 34000, DGO, Mexico; lavallec@outlook.com (J.L.-C.); carreonburciaga55@hotmail.com (R.G.C.-B.); ronellbologna@hotmail.com (R.B.-M.); 3 Molecular Biology Department, School of Dentistry, Universidad Autónoma de Baja California, Mexicali 21040, Mexico; nserafin@uabc.edu.mx; 4 Department of Health Care, Xochimilco Unit, Universidad Autónoma Metropolitana (UAM) Xochimilco, Mexico City 04960, Mexico; nmolinaf@hotmail.com; 5 Molecular Pathology Area, School of Dentistry, Universidad de la República, Montevideo 11600, Uruguay

**Keywords:** oral squamous cell carcinoma, drug resistance, epigenetics

## Abstract

This manuscript provides an update to the literature on molecules with roles in tumor resistance therapy in head and neck squamous cell carcinoma (HNSCC). Although significant improvements have been made in the treatment for head and neck squamous cell carcinoma, physicians face yet another challenge—that of preserving oral functions, which involves the use of multidisciplinary therapies, such as multiple chemotherapies (CT) and radiotherapy (RT). Designing personalized therapeutic options requires the study of genes involved in drug resistance. This review provides an overview of the molecules that have been linked to resistance to chemotherapy in HNSCC, including the family of ATP-binding cassette transporters (ABCs), nucleotide excision repair/base excision repair (NER/BER) enzymatic complexes (which act on nonspecific DNA lesions generated by gamma and ultraviolet radiation by cross-linking and forming intra/interchain chemical adducts), cisplatin (a chemotherapeutic agent that causes DNA damage and induces apoptosis, which is a paradox because its effectiveness is based on the integrity of the genes involved in apoptotic signaling pathways), and cetuximab, including a discussion of the genes involved in the cell cycle and the proliferation of possible markers that confer resistance to cetuximab.

## 1. Introduction

Cancer is a complex and heterogeneous disease that is regulated at multiple levels and is associated with high mortality and morbidity indexes. Head and neck squamous cell carcinomas (HNSCCs) are genetic disorders that are related to environmental risk factors, especially excessive alcohol and tobacco consumption [1]. Changes in the chromatin landscape via mutations, deletions, amplifications of the genomic sequence, and epigenetic perturbations lead to abnormal alterations in gene expression; the effect of defective epigenetic mechanisms has been extensively studied in HNSCC, which is the seventh most frequent cancer, with a global incidence of more than half a million annual cases [1,2]. Particularly, oral squamous cell carcinomas (OSCCs) are the prevalent forms of HNSCC and represent approximately 90% of all tumors in this region. The high mortality associated with OSCC is related mainly to the locoregional advancement of the disease [2,3,4].

Although significant improvements have been made in achieving local control of the disease and increasing the survival rate of patients with primary malignant oral tumors via surgical intervention, physicians face yet another challenge—that of preserving oral functions, such as articulation, mastication, and deglutition, and retaining visual aesthetics for improving HNSCC patients’ quality of life. Effective treatment for advanced HNSCC (T3 and T4), but not resectable tumors, involves the use of multidisciplinary therapies, such as multiple chemotherapies (CTs) and radiotherapy (RT).

CT regimens for head and neck area tumors include the use of combinations of chemotherapeutic agents such as cisplatin and platinol (CDDP) and PF therapy (cisplatin + fluorouracil), involving the use of an antagonist of the pyrimidine metabolism of 5-fluorouracil (5-FU) [5,6]. Recently, the chimeric mouse–human monoclonal Epidermal Growth Factor Receptor (EGFR) antibody Cetuximab (Erbitux, Merck KGaA, Darmstadt, Germany) was approved by the Food and Drug Administration (FDA) for the treatment of locally advanced HNSCC, in combination with radiation, as well as for recurrent/metastatic diseases, together with other chemotherapeutics [7].

The most used treatment strategy for advanced HNSCCs is CDDP at a dose of 100 mg/m^2^ plus RT [8,9,10]. However, the efficacy of CDDP-based treatment can be limited, as patients acquire intrinsic drug resistance [11,12]. The treatment sensitivity to antineoplastic drugs is correlated with the individual characteristics of patients and genetic differences among clonal cells that belong to the same tumor, a phenomenon called tumor heterogeneity [12]. Importantly, the interactions among these factors generate a limited combination of genetic alterations, and consequently, the sensitivity and resistance of the neoplastic cells to the treatment may vary among individuals [8,9,10,11,12].

**Figure 1:**
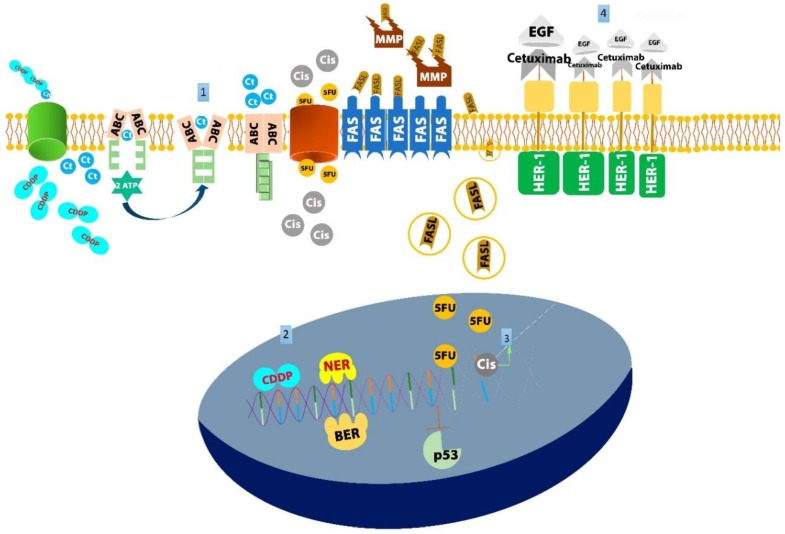
The principal molecular markers according to drug resistance mechanism-based groups in head and neck squamous cell carcinomas (HNSCCs). (**1**) Reduced concentration of antineoplastic drugs in cancerous cells. The family of ATP-binding cassette (ABC) transporters mostly includes P-glycoproteins (P-gp), which intracellularly bind to cytostatic agents and promote their exocytosis via ATP hydrolysis and conformational changes in the protein. Extracellularly, alterations in plasma membrane proteins may also decrease drug permeability. Expression or overexpression of the ABC genes encoding *MDR1*, *MRP1*, *MRP2*, and *BCRP* is involved in oral squamous cell carcinoma (OSCC) chemotherapeutic resistance. (**2**) Increased DNA reparation ability of tumor cells. An increase in the tolerance to DNA damage because of highly efficient DNA repair machinery may be caused by the gene encoding components of the nucleotide excision repair and base excision repair (NER and BER) complexes. Polymorphisms in DNA repair genes may be used for predicting favorable clinical results in patients with HNSCC. (**3**) Enhanced tumor survival and routes of dissemination. FasL is upregulated in cells treated with cisplatin and 5-FU, which induce programmed cell death. Alterations in the gene encoding *p53* silence matrix metalloproteinases (MMPs) overexpression, which has been associated with the survival and dissemination of tumors and drug resistance. (**4**) Inactivation of antineoplastic drugs. Increasing evidence suggests that EGFR ligands influence the response to EGFR-targeted therapy and might be useful as predictive biomarkers. The autocrine growth factor production might compete with blocking antibodies for binding to EGFR and thereby reduce their effectiveness.

The antineoplastic drug resistance mechanisms are classified into four groups: (1) reduced concentration of antineoplastic drugs in cancerous cells; (2) increased DNA reparation ability of tumor cells; (3) enhanced tumor survival and routes of dissemination; and (4) the inactivation of antineoplastic drugs [12] (Figure 1).

Ethnic differences, physical characteristics (age, weight, and gender), and genomic alterations related to the survival and perpetuation of tumor cells are considered when designing personalized therapeutic options. This also includes the evaluation of genomic alterations, punctual mutations, and changes in the gene copy number as they affect the longevity of neoplastic cells and the survival rates of neoplastic cells. Thus, cancer diagnostics should be based on the genomic information for arriving at correct therapeutic decisions [13].

## 2. Reduced Concentration of Antineoplastic Drugs in Cancerous Cells 

Palliative therapies, such as RT, are focused on reducing the local symptomatology and improving the quality of life of patients [14]. However, CT therapy combined with multiple cytostatic agents and RT can lead to “tumor chemoresistance” or “multiple drug resistance” (MDR), which may change according to the type of treatment [15].

The development of MDR can lead to a resistance to other drugs that are not structurally related. MDR can be understood through different biological factors and it is frequently linked with drug efflux. There is increasing evidence regarding the regulated entrapment of the transporter proteins expressed in the cell membrane which are responsible for drugs transporting through the plasma membrane to the outside of the cell. The ATP-Binding Cassette (ABC) Family comprises transporters that can drive chemotherapeutic agents to the outside of the cell in order to resist their cytotoxic effects. Additionally, ABC transporters play an important role in importing and exporting nutrients and molecules, thereby representing an important obstacle in oncological therapies [16].

### ATP-Binding Cassette Transporter (ABC)

P-glycoprotein (P-gp), a product of the gene *ABCB1* (*MDR1*), is an ABC transporter associated with MDR, and it has been demonstrated to provide resistance to multiple chemotherapeutic drugs [16]. The *MDR1* gene encodes a P-gp transmembrane segment that is used to evacuate different drugs. The mechanism starts when the drug molecule binds to the P-gp cytoplasmic domain; then, the protein uses ATP hydrolysis to open into the extracellular space and evacuate the drug molecule [17]. The overexpression of P-gp may lead to a resistance 100 times higher than that of a normal cell [18].

Previous studies have indicated that P-gp expression is involved in the MDR of head neck cancer (HNC) [19,20,21,22], although the process that triggers P-gp expression is not clear. Differences in P-gp expression can occur in clonal cells of the same tumor (cellular heterogeneity), as the activated production of P-gp can be inherited or acquired [19,23,24]. However, the P-gp production’s relation to the MDR phenotype is not completely understood, and MDR may be the result of intrinsic or acquired resistance [25,26,27].

Furthermore, it has been demonstrated over the last few decades that P-gp transference to the cell is through microvesicles (MVs). In malignant tumors there exist increased numbers and transport of different types of MVs that are related to tumor progression processes and carcinogenesis associated with an MDR developmental factor [28]. Although this has been demonstrated in different tumors, there is no recent proof of these events in head and neck tumors.

Friedrich et al. analyzed the *MDR1*, *MRP1*, and *BCRP* genic expressions in primary SCCs and observed that *MDR1* and *MRP1* were co-expressed. However, *BCRP* expression was not *MDR1*-dependent and did not show an overregulation with normal *MRP1* expression. Certain tumors showed similar expression patterns of *MDR1* and *BCRP*, indicating that *BCRP* overexpression is related to *MDR1* and that the patient survival can be influenced by the altered expression of at least one of the genes implicated in the chemotherapeutic resistance [29].

Nakamura et al., using CDDP-sensitive and -resistant cell lines (H-1 and H-1R), observed that the resistant cells had a high *MDR1* and low *MRP1* expression. Thus, the opposite *MDR1* and *MRP1* expression pattern could be related to CDDP resistance in cell cultures [30].

Suzuki et al. observed that *MDR1* and *ERCC1* (genes discussed in the section on nucleic acid repair/base excision repair (NER/BER) genes) were not expressed in any single cell clone from the primary SCC tumors, although *MRP1* and *MRP2* were expressed in all single cell clones [12].

## 3. Increased DNA Reparation Ability of Tumor Cells 

Most of the nonresectable treatments of advanced HNSCCs involve cisplatin along with radiotherapy; cisplatin binds to DNA, forming adducts and facilitating the intracellular accumulation of free radicals. It has been discovered that the expression of different Single Nucleotide Polymorphisms (SNPs) are related to different toxicity levels of this drug. In HNSCCs, there exist polymorphic alterations associated with DNA mismatch repair protein (MMR) pathways that may enhance secondary effects in response to cisplatin and RT [31].

It is possible that these alterations are associated with chemotherapy resistance, leading to the modification of treatment procedures in these types of carcinomas.

### Nucleotide Excision Repair (NER)/Base Excision Repair (BER)

The DNA repair system has the important function of protecting the genomic material from presenting any mutations so that it maintains its total integrity. Nucleotide Excision Repair (NER) is a DNA reparation pathway, and its mechanism of action is accomplished by removing DNA that has been damaged, mainly by chemical carcinogens. Most of the NER genes are polymorphic, and there are reports indicating a relationship between distinct NER gene polymorphisms and tobacco risk factors associated with lung, head and neck, and breast cancer [32].

Base Excision Repair (BER) repairs DNA bases injured by mutagenesis and toxicity processes, both of which are important factors in the etiology and treatment of cancer since chemotherapeutic agents induce genotoxic damage to DNA bases, act as BER substrates, and cause DNA damage repair. BER affects the cellular response to ionizing radiation, which modifies the DNA structure and then recognizes the damage in need of reparation. Additionally, BER is implicated in the cytotoxicity of 5-FU, a drug whose metabolites are integrated into both DNA and RNA [33].

Polymorphisms in the genes encoding these enzymes were studied by Quintela et al. in the blood of patients with HNSCC [34]. Although a polymorphism in *ERCC1* (C8092A) has been reported to affect the mRNA stability and alter the DNA reparation capability, no differences were detected in the *ERCC1* sequence of HNSCC survivors and patients undergoing cisplatin therapy. However, Ameri et al. recently showed that a high level of *ERCC1* expression in patients with HNSCC did not show any correlation with the chemotherapeutic response. Therefore, it has been suggested that the decrease of *ERCC1* expression may be related to increased chemoradiation sensitivity and an improved clinical outcome. Thus, it is not surprising that *ERCC1* is a frequently evaluated marker in HNSCCs [35,36].

Two single nucleotide polymorphisms (SNPs) in *XPD*/*EDRCC2* (Asp312Asn and Lys751Gln) and one in *XRCC1* (Arg399Gln) have been associated with the suboptimal capability of DNA reparation. However, unlike the case with *ERCC1*, Quintela et al. concluded that all polymorphic variants of *XPD* and *XRCC1* provide a better prognosis and response to chemotherapy, and each polymorphic variant provides a 2.1–3 times increase in the probability of achieving a complete response to treatment [34].

## 4. Enhanced Tumor Survival and Routes of Dissemination 

CDDP and 5-FU are cytotoxic chemotherapeutic agents that affect HNSCC cell lines, improving radiation side effects. The combination Taxotere-Cisplatin-5-FU (TPF) induces apoptosis and necrosis in cell lines and, along with other chemotherapeutics, induces the downregulation of the cell proliferative rate targeted by Ki67 and Bcl-2 [37,38].

### 4.1. TP53

Tumor protein *53* (*TP53*) encodes a nuclear transcription factor known as tumor protein p53, which acts as a tumor suppressor. p53 regulates the cell cycle checkpoints and DNA repair but is frequently associated with apoptosis. Additionally, it participates in the repair process in response to damaging factors, including chemicals, radiation, and ultraviolet rays from sunlight. If the DNA is mutated or damaged and cannot be repaired, p53 transmits a signal which triggers cell apoptosis and prevents cells from dividing and developing into tumors [39].

*TP53*’s loss of heterozygosity and exon mutations and the presence of anti-p53 antibodies in the plasma are considered independent predictive factors of a low response to neoadjuvant chemotherapy with 5-FU/cisplatin [39].

Tumors that show an overexpression of p53 are resistant to CT and RT treatments, causing a decrease in the survival rates, probably because of its relation to induction and tumor progression, as it is associated with gene mutations and alterations of cell functions [40].

### 4.2. Fas/FasL

Apoptosis via the extracellular TNFRSF6/TNFSF6 (Fas/Fas ligand (FasL)) pathway induces apoptosis in the presence of genotoxic insults [41]. FasL and its receptors (Fas, CD95) are part of the receptor family of Tumor Necrosis Factors (TNFs), which participate in the immune system as important regulators. The interaction between FasL and Fas leads to apoptosis. In normal circumstances, the overexpression of Fas in T-cells proves to be a mechanism that limits the immune response, participating in immune and peripheral homeostasis, and eliminating clonal activated T-cells [42]. It is well known that extrinsic apoptosis is triggered by the TNF family enzymes, including Fas/FasL; FasL is expressed in most tumors, including oral cancer, and is related to resistance to apoptosis induction. Most of the chemotherapeutic agents act as apoptosis inducers in HNSCCs, in which the Fas/FasL signaling pathway may play an important role in chemoresistance through extracellular Matrix metalloproteinase (MMP) 7, due to the fact that drugs such as doxorubicin and oxaliplatin may lead to the upregulation of MMP7. Thus, MMP7 causes the generation of soluble FasL (sFasL) [43].

Few studies have indicated that polymorphisms in certain MMPs are independent risk factors for the development of chemotherapy resistance. Blons et al. observed a significant correlation between MMP3 polymorphism and the response to chemotherapy in French patients with HNSCCs. They found that subjects with a poorly transcribed 6A allele showed better responses to 5-FU-cisplatin combined therapy [44].

### 4.3. Complement System

Located at the cell surface exist the regulator proteins of the complement system—CD46, CD55, and CD59—which control the complement activation and its different pathways. CD55 (decay-accelerating factor) is a regulatory molecule of the complement system that, along with CD59 (protectin) and CD46 (membrane cofactor), prevents the intrinsic attack of C3 convertase, the key enzyme responsible for triggering membrane attack and cellular disintegration [45]. This mechanism is called cellular death by complement and is considered an independent process of caspase activation that is associated with the reactive oxygen species (ROS) formation [46]. Overexpression of the mentioned regulator proteins of the complement system is related to the prevention of complement-dependent cytotoxicity in cancer cells. It is important to establish that the overexpression of CD55 may promote tumor initiation by the inhibition of natural-killers cell (NK) growth. Additionally, this may contribute to the downregulation of the complement system’s activity, facilitating tumor progression. Furthermore, when CD55 binds to CD97, it stimulates migration, invasion, and metastasis, although its role is not well defined in HNSCCs [47]. As mentioned in the section on MDR genes, Nakamura developed an assay for analyzing microarrays, in which he included genes related to apoptosis, for studying an alternative mechanism of MDR. Among the genes expressed differentially in either the sensitive or resistant colonies, CD55 was overexpressed in the H-1R colony [30].

## 5. Inactivation of Antineoplastic Drugs

The Epidermal Growth Factor (EGF) and its receptor, the Epidermal Growth Factor Receptor (EGFR), are involved in cancer patients with poor prognosis, of which 80–90% are associated with HNSCCs. This indicates that the overexpression of the complex is related to increased cell proliferation, migration, and resistance to apoptosis. Cetuximab is a drug involved in the interference of natural EGFR ligands, as well as in the induction of endocytosis receptors, blocking their signaling [48].

Cetuximab is a chimeric monoclonal immunoglobulin IgG1 which binds to the extracellular domains of EGFR. This drug blocks the activation of the receptor by preventing the tyrosine kinase-mediated phosphorylation of the protein, leading to antibody-dependent cellular cytotoxicity. Therefore, the host’s immune system may attack cells covered with the antibody bound to EGFR. The downstream main effects of cetuximab are the promotion of apoptosis, the inhibition of cell cycle progression, tumor cell invasion, and angiogenesis [49].

Cetuximab has been used in patients with HNSCC, showing an improvement in those with recurrences and metastasis, increasing survival in conjunction with RT and another CT. However, there has been a significant rate of recurrence after treatment was found to be associated with drug resistance mediated by EGFR [49,50,51,52].

### 5.1. HER1

EGFR is overexpressed in approximately 30% of all human epithelial tumors, including HNSCCs, of which nearly all tumors exhibit EGFR overexpression [53]. EGFR is a transmembrane tyrosine kinase cell surface receptor that belongs to the ErbB2 family.

The receptor family ErbB is formed by four types of receptors: EGFR (also known as ErbB/HER1), ErbB-2 (neu, HER2), ErB-3 (HER3), and ErB-4 (HER4) [54]. There exist 11 ligands that interact with ErbB receptors. According to their binding type, they are classified in three groups: (a) bound to EGFR (EGF, Transforming Growth Factor-α (TGF-α), amphiregulin (AREG), and epigen (EPGN)); (b) bound to EGFR and ErbB4 (Beta Cellulin (BTC), Heparin-Binding EGF (HB-EGF)); and (c) Neuregulin (NRG). These ligands are secreted by tumor cells and participate in autocrine and paracrine stimulation [54,55].

There are some studies that propose EGFR ligands as predictive biomarkers for EGFR-targeted therapies. However, to date, it has not been concluded which of the EGFR ligands definitively predict the efficacy of these therapies in patients with HNSCC [56,57,58,59,60].

Ansell et al. studied these ligands along with EGF in three tongue cancer cell lines. Their results suggest that even though EGF expression is low in cancer cell lines, it might be critical for maintaining tumor cell proliferation, and this might be also sufficient to confer resistance to cetuximab [7]. Other EGFR ligands that have a lower affinity to EGF, such as TGF-α, HB-EGF, AREG, BTC, and EPGN, may stimulate the growth, invasion, and metastasis due to their dysregulation in cancer, which promotes the higher tumor survival through autocrine or paracrine stimulation. Therefore, autocrine growth factor production might compete with blocking antibodies and prevent them from binding to EGFR, thereby reducing their effectiveness [61].

### 5.2. Aurora Kinase A and B

Aurora kinases A and B (AurkA and AurkB) are highly conserved serine/threonine kinases that play essential and distinct roles in mitosis. AurkA is required for the assembly of the mitotic spindle and accumulates on centrosomes at the spindle poles during prophase until metaphase [62]. Furthermore, the upregulation of AurkA leads to abnormal centrosome numbers and the induction of aneuploidy [62,63,64]. This overexpression is associated with HNSCC and promotes cell proliferation, tumor progression, and metastasis [65]. Previous reports showed that the EGFR and AurkA protein levels were elevated in tumor tissue, which represents a risk group with a poor disease-free survival [66]. Additionally, both proteins and EGFR share downstream signaling pathways, and each by itself represents a potential therapeutic target in HNSCCs.

Our search found one study which provides in vitro evidence for the predictive value of AurkA polymorphism in the efficiency of cetuximab treatment. AurkA genotypically homozygous HNSCC cells respond to cetuximab monotreatment, whereas heterozygous cells do not. Moreover, cetuximab resistance can be overcome by siRNA-based AurkA/B knockdown in vitro. The combination of cetuximab and anti-AurkA/B targeting in HNSCC cells ameliorates any polymorphism-related difference and increases the treatment efficiency, independent of the Aurora kinase genotype [67]. Table 1 explains the expression of molecular markers and their participation in multidrug resistance.

## 6. EMT-CSC Involved in Treatment Resistance

Several solid tumors, including HNSCCs, have the capacity to initiate and maintain tumor growth and present with recurrences. These tumor characteristics are related to small cell populations known as Cancer Stem Cells (CSCs), which have the capability of self-renewing and maintaining a differentiated cell line, as well as preserving some pluripotent phenotypes capable of producing tumors comprising heterogenic cell populations, which is related to tumor invasion, metastasis, and therapeutic resistance with recurrent tumors after treatment [68].

In HNSCCs, subpopulations of CSCs can be identified by high expression levels of hyaluronan receptor CD44. It is important to highlight that CSCs are closely related to Epithelial–Mesenchymal Transition (EMT), in which the interaction of EMT-CSCs is probably related to invasion processes and tumor progression [69].

HNSCCs present different cell subpopulations within the tumor that are different from each other, which is associated with tumorigenicity and metastatic potential [70]. Tumors that present with therapy resistance may originate from CSCs or from tumor cells with the Epithelial–Mesenchymal Transition phenotype which loses polarity and cell–cell contacts, acquiring the capability to migrate towards the mesenchyme with a migratory mesenchyme phenotype [68,70]. It is important to mention that Snail is a transcription factor with zinc fingers that plays an important role in EMT and may suppress epithelial markers, such as E-cadherin, and upregulate mesenchymal markers. Snail is associated with transcription factors related to important regulatory processes in invasion, metastasis, and a bad prognosis, in addition to its relation to motility and apoptosis resistance. Additionally, cells with the EMT phenotype may acquire similar properties to CSCs and thereby achieve resistance to antineoplastic treatments [68].

EMT is a reversible cell process which is induced mainly by the paracrine mechanism of small molecules related to fibroblasts associated with the tumor, where Tumor Growth Factor β1 (TGF- β1) is one of the most important mediators [71,72]. TGF-β1 interacts with the similar TGF-β1 type I and II, tyrosine-kinase receptors that activate the Smad 2 and 3 signaling pathways, forming complexes with Smad 4 that act as transcription factors related to Snail and the activation of EMT [73].

Previously, EGFR has been described as a therapeutic target of agents such as cetuximab and Erlotinib. Several mechanisms are related to cetuximab resistance, which may be also associated with different lines of cell subpopulations of HNSCCs. Therefore, EGFR inhibition is related to morphological and molecular changes and decreasing cell proliferation, however, no relationship is established with intense cell death induction. These features may be associated with a reduction in the CD44 expression and the initiation of cell differentiation of CSCs, after which tumors may attain therapeutic resistance characteristics [69].

Several studies indicate that monotherapy treatment with cetuximab is related to a low therapy response in advanced HNSCCs. Otherwise, if cetuximab is combined with RT or platin/fluorouracil-based therapies, the treatment response improves, including in patients with metastatic HNSCCs, where monotherapy with Erlotinib has a lower rate response, improving when it is combined with cisplatin or RT [69,74]. It is possible that the treatment response rate is related to the capability of EGF to initiate EMT and inhibit epithelial differentiation, initiated by Epithelial-CSCs (Epi-CSCs) and EMT-CSCs [69,75]. Thus, it is possible that CSCs’ control over HNSCCs may be related to therapies focused on the control of tumor growth, development, and therapy resistance [69].

## 7. Independent Molecular Markers of the Resistance Mechanism of Antineoplastic Drugs

Cytochrome P450 (CYP) is a large superfamily of integral membrane conserved proteins present in animals, plants, and microorganisms. The CYP isoenzyme superfamily comprises 57 CYP genes and 58 pseudogenes arranged into 18 families and 43 subfamilies in humans. They are heme-containing proteins that catalyze the oxidative metabolism of many structurally diverse drugs and chemicals [76].

CYPs are grouped into families and subfamilies according to the similarity of their amino acid sequences. The enzyme code starts with CYP, followed by a designating number for the family [77]. Then, a letter for the subfamily is added, ending with an individual number for the gene. With regard to the drug metabolism, phenotypes for CYP polymorphism range from ultrarapid to poor metabolizers. The latter may lead to drug accumulation and intoxication, while rapid metabolizers require a higher dose to accomplish the desired effect [76].

Most of the studies in patients with HNSCC have demonstrated the association between no-response and poor response treatment. In cases with poor metabolizer genotypes, CYP2D6 alone or in combination with CYP2C9 or CYP2C19 (CYP2C19*2 and CYP2C19*3), particularly in those with a CYP2C19*2 genotype or both, may have a synergistic role in modulating treatment response. At the same time, CYP2A6 was found to modulate the treatment outcome in HNSCC cases, and the treatment response was poor, particularly in cases with at least one deletion allele of CYP2A6 [78].

Although these enzymes may not have a direct role in the metabolism of cisplatin or the majority of the chemotherapeutics (except CYP2C9 to a small extent), these drug-metabolizing cytochrome P450 enzymes are involved in the metabolism of several of the supportive care drugs used in chemotherapy [76,77,78].

## 8. Future Perspectives

Treatment in advanced-metastatic HNSCCs is frequently treated with multidisciplinary therapy with the purpose of improving prognosis. However, resistance to this type of therapy may be present in this disease [79].

The suggestion of personalized therapy, based on the identification of genetic alterations that endow tumor cells with drug resistance or render them susceptible to chemotherapy, appears to be a better option for selecting the correct chemotherapeutics with minimal side effects.

Considering the groups based on antineoplastic drug resistance mechanisms, the genes included in this review can be classified as follows. Group 1: ATP binding cassette, Group 2: NER/BER, Group 3: *TP53*, Fas/FasL and the complement system, and Group 4: EGF, AurkA, and AurkB. 

Another important association in antineoplastic treatment resistance, including immunotherapy resistance, involves CSCs and the mesenchymal phenotypes of tumor cells in HNSCCs. These are characteristics of aggressive tumors that provide resistance to antineoplastic treatments and must be analyzed prior to oncological treatment in order to establish combined and/or personalized therapies to overcome the resistance of these cells, thereby decreasing the treatment morbidity. However, it is possible that these cellular events may associate through adaptation pathways to other adaptative processes, such as telomeric regulation or alterations in cell cycle controls, that cause resistance to treatments.

Another gene has recently been postulated as a target to inhibit resistance to antineoplastic drugs in HNSCC. Survivin, an antiapoptotic molecule abundantly expressed in most human neoplasms, has been reported to contribute to cancer initiation and drug resistance in a wide variety of human tumors. It has been proposed that the efficient downregulation of survivin can sensitize tumor cells from squamous cell carcinomas to various therapeutic interventions (Group 3 of the drug resistance mechanisms) [80]. Other perspectives must be considered, such as the tumor site, which is an important variable to consider in the drug resistance mechanisms [81].

It has been seen that polymorphic alterations of the cytochrome p450 family are related to drug therapy resistance in HNSCC patients. However, more studies are required to establish the resistance mechanisms to antineoplastic drugs.

## 9. Conclusions

We have discussed the roles of various genes involved in the response of tumor cells to anticancer therapies, which assists in predicting the efficacy of the therapies. However, the percentage of cellular heterogeneity in tumors should also be considered for identifying the genes involved in resistance to antineoplastic therapy or the efficacy of any treatment. In addition, novel tools, such as the genome-wide association study (GWAS), must be used to associate more than one gene simultaneously with a trait or phenotype, for example, resistance to antineoplastic therapy in patients with HNSCC.

## Figures and Tables

**Table 1:** The expression of molecular markers and their participation in multidrug resistance in head and neck squamous cell carcinoma.

Author	Year	Genes	Methodology	Conclusions
Reduced concentration of antineoplastic drugs in cancerous cells.				
**Friedrich**	2004	*MDR1*, *MRP1* and *BCRP*	Gene expression in primary SCC using IH and PCR.	*MDR1* and *MRP1* are co-expressed; *MDR1* and *BCRP* are not co-dependent. Patient survival can be influenced by the altered expression of at least one of the genes implicated in chemotherapeutic resistance.
**Nakamura**	2005	*MDR1*, *MRP1*	Expression levels in CDDP-resistant/sensitive cell lines using in-house cDNA microarray (2021 genes originated from normal oral tissue, primary oral cancer, and oral cancer cell lines) and PCR.	Resistant cells have high *MDR1* and low *MRP1* expression.
**Suzuki**	2010	*MDR1*, *MRP1* and *MRP2*	Gene expression analysis of single cell clones dissociated from primary tumors using PCR.	*MDR1* was not expressed in any single cell clone from primary SCC tumor, although *MRP1* and *MRP2* were expressed.
Genes involved in DNA repair				
**Quintela**	2006	*XPD*, *ERCC1* and *XRCC1*	SNP detected using RFLP in DNA from peripheral lymphocytes of HNSCC patients.	The accumulation of polymorphic variants increases the probability of achieving a complete response.
**Ameri**	2016	*ERCC1*	Expression status determined using PCR in tumor samples.	Tumor samples with high *ERCC1* expression showed no response to induction chemotherapy.
Enhanced tumor survival and routes of dissemination				
**Cabelguenne**	2000	*TP53*	Gene status (mutations, allele loss) detected using PCR amplification in tumor samples.	P53 status may be a useful indicator of responding to neoadjuvant chemotherapy in HNSCC.
**Blons**	2004	*MMP3*	*MMP1*, *MMP3*, and *MMP7* polymorphisms detected using PCR in tumor samples and blood.	A significant correlation between *MMP3* polymorphism and response to chemotherapy.
**Nakamura**	2005	*CD55*	Expression levels in CDDP-resistant/sensitive cell lines using in-house cDNA microarray (2021 genes originated from normal oral tissue, primary oral cancer, and oral cancer cell lines) and PCR.	*CD55* was overexpressed in the H-1R colony.
Inactivation of antineoplastic drugs				
**Ansell**	2016	*AR*, *EPR* and *EGF*	Response was evaluated by adding recombinant human proteins or siRNA-mediated downregulation of endogenous ligand production.	The amount of EGF strongly influences the tumor cell proliferation rate and response to cetuximab treatment. Proposed EGF as a potential predictive biomarker
**Pickhard**	2014	*AurkA* and *AurkB*	IH in tissue samples.	Provide evidence that AurkA genotypically homozygous HNSCC cells respond to cetuximab monotreatment, whereas heterozygous cells do not.

*MDR1*: Multidrug resistance 1; *MRP1*: Multidrug resistance protein 1; *BCRP*: Breast cancer related protein; CDDP: Cisplatin and platinol; SCC: Squamous cell carcinoma; IH: Immunohistochemistry; PCR: Polymerase chain reaction; *XPD*: Xeroderma pigmentosum protein; *ERCC1*: Excision repair cross-complementing group 1; *XRCC1*: X-ray repair cross-complementing protein 1; SNP: Single nucleotide polymorphism; RFLP: Restriction fragment length polymorphism; HNSCC: Head and neck squamous cell carcinoma; *MMP* 1, 2 and 7: Matrix metalloproteinase 1, 2 and 7; H-1R: CDDP-resistant cell line.
